# Listening to the screaming whisper: a voice of mother caregivers of children with autistic spectrum disorder (ASD)

**DOI:** 10.1080/17482631.2018.1479585

**Published:** 2018-06-21

**Authors:** Kiboum Kim, Junhyoung Kim, Alison Voight, Minjoon Ji

**Affiliations:** a Department of Human Services Studies, Winston Salem State University, Winston Salem, NC, USA; b Department of Health and Human Performance, Texas State University, San Marcos, TX, USA; c Department of Recreation Park and Tourism Studies, Indiana University, Bloomington, IN, USA; d Department of Sport Science, Kyungnam University, Changwon, South Korea

**Keywords:** Autistic disorder, health, leisure constraint, mother caregivers

## Abstract

**Purpose:**This is a gap in knowledge related to leisure behaviours among mothers who have children with autistic spectrum disorder (ASD). This study intended to understand leisure behaviours associated with leisure constraints among these mother caregivers. **Method:** Using a series of semi- structured interviews, a total of 12 participants engaged in this study. **Results:** Our findings consisted of two sections: (1) leisure negotiation and (2) leisure constraints which are (a) a lack of time for themselves, (b) constant attention, and (c) difficulty in finding a helper. **Conclusions:** This study found that participants modified their leisure patterns as they sought to provide more recreational opportunities for their children. It suggests that participants negotiated their leisure choices and developed family adaptability related to family leisure in order to suit the desires and needs of their child with ASD.

## Introduction

According to American Association of Retired Persons (AARP, 2015), there are approximately 37 million adults in the U.S.A. who regularly provide unpaid care for a relative or a family member. Parent caregivers are a group of individuals who are committed to providing physical, emotional, and social support for their children with special healthcare needs to an extent that often exceeds what would typically be expected of parents. These parents retain primary responsibility for providing basic needs such as protection and nutrition. They also take on additional responsibilities related to education and healthcare. Prior studies have suggested that parent caregivers often accommodate their children’s needs by limiting their own personal life opportunities. They focus more on their children rather than on themselves (Abbeduto et al., 2004; Weiss, 2002). As a result, these parents are likely to exhibit psychological stress and experience lower levels of perceived well-being and quality of life.

In particular, mother caregivers who have children with complex disabilities such as Autistic Spectrum Disorder (ASD) may experience increased psychological burdens and stress. Substantial research suggests that children with ASD exhibit communication challenges with impaired ability to interact with others, repetitive and restricted stereotyped behaviours, and socially deviant behaviours (Gray, 1994; Norton & Drew, 1994; Schreibman, Heyser, & Stahmer, 1999). Management of these behaviours associated with ASD often demands high physical and emotional investment from mother caregivers. Compared to parent caregivers who have children with other disabilities, mothers of children with ASD have been reported to have higher levels of stress and psychological problems (Rivers & Stoneman, 2003; Strain & Schwartz, 2001).

Participation in leisure activities can be an important strategy to reduce life stress, improve family relationships, and increase life satisfaction for mother caregivers who have children with ASD. Empirical studies have provided evidence that leisure activities have significant health benefits through positive social relationships, resulting in improved physical function and psychological wellbeing (Coleman & Iso-Ahola, 1993; Iwasaki, 2002; Kleiber, Hutchinson, & Williams, 2002). However, mothers of children with ASD are often deprived of possible health benefits from leisure participation. Various scholars have suggested that mother caregivers who have children with ASD have limited opportunity to pursue their personal leisure interests or engage in leisure and social activities compared to parents who have children without disabilities (Mugno, Ruta, D’Arrigo, & Mazzone, 2007; Rivers & Stoneman, 2003; Wilson, Moore, Rubin, & Bartels, 1990; Weinblatt & Navon, 1995). Furthermore, the prevalence of social stigma towards persons with disabilities in the general public and the lack of social support for parents of children with disabilities often exacerbate limitations to their leisure participation and discourage mothers of children with ASD from getting involved in daily leisure activities without their children (Corman, 2007; Norton & Drew, 1994).

Social stigma, lack of social support, and limited opportunity are only several of various barriers to participation in leisure activities for mothers of children with ASD. In fact, these mother caretakers may experience a high degree and frequency of leisure constraints. Jackson (1988) has defined the concept of leisure constraints as things or circumstances “that inhibit people’s ability to participate in leisure activities, to spend more time doing so, to take advantage of leisure services or to achieve a desired level of satisfaction” (p. 203). Such leisure constraints can negatively affect life satisfaction and quality of life among mother caregivers who have children with ASD. To facilitate leisure engagement of these mother caregivers and expose them to benefits of leisure participation, leisure service providers and recreational therapists must first explore existing leisure behaviours and constraints of this population.

Prior studies have primarily focused on leisure behaviours among children with ASD and other disabilities (Mactavish, McKay, Iwasaki, & Betteridge, 2007; Scholl, McAvoy, & Smith, 2000). Considering the gap in knowledge related to experiences of mothers of these children, the purpose of this study was to explore leisure behaviours associated with leisure constraints of mothers who have children with ASD. Information gained through this study will contribute to knowledge on how to minimize leisure constraints experienced by mother caregivers.

## Literature review

### Life experiences of mother caregivers for children with ASD

Numerous studies have suggested that mother caregivers experience significant psychological stress due to their physical, emotional, and financial support for their children with ASD (Abbeduto et al., 2004; Weiss, 2002). According to Rivers and Stoneman (2003), mothers who have children with ASD are reluctant to seek professional help from other healthcare providers as they believe other providers cannot do as much as they can. In addition, mothers who have children with ASD often encounter feelings of guilt when they rely on the help of others in the care of their children. Prior studies have also demonstrated that mothers who have children with ASD tend to experience financial challenges because they invest their money in various support services such as special education and specialized therapies to improve their children’s functional abilities (Abbeduto et al., 2004; Rivers & Stoneman, 2003). Each of these specific stressors is further evidence that mother caretakers of children with ASD have high levels of general psychological stress.

Mothers who have children with ASD are likely to experience mental health problems beyond high stress levels (Parkes, Caravale, Marcelli, Franco, & Colver, 2011; Raina et al., 2005). Compared to mother caregivers of children with other disabilities, it has been reported that caregivers of children with ASD have higher levels of depression and anxiety but lower levels of psychological wellbeing (Benson & Karlof, 2009; Ekas, Whitman, & Shivers, 2009). Depression is a particularly prevalent psychological issue among mothers of children with ASD (Parkes et al., 2011). It has been reported that mother caretakers with depression exhibit negative parenting behaviours and experience other psychological problems such as anxiety and isolation (Parkes et al., 2011; Raina et al., 2005). However, other studies have shown that caregivers of children with ASD demonstrate effective coping strategies and adaptation to their life circumstances that result in family resilience (Jones & Passey, 2005; McCubbin, McCubbin, & Thompson, 2001). They suggested that mothers of children with ASD experienced family resilience, expanded social networks, and developed a sense of spirituality. In addition, Plumb (2011) has found that there is a negative relationship between family resilience and parental distress among parents of children with ASD. These studies stress the importance of family adaptation to adversity and family resilience.

### Leisure constrains experienced by mother caregivers

Leisure plays an important role in our society as it contributes to physical, social, and psychological benefits. Mothers of children with disabilities can alleviate psychological distress, foster social interactions, and promote psychological wellbeing through leisure engagement. Research on leisure has shown that mothers of children with disabilities can use participation in leisure activities as a coping strategy and coping resource (e.g., Bayat, 2007; Gray, 2006). For example, Kuhaneck, Burroughs, Wright, Lemanczyk, and Darragh (2010) have explored coping strategies used by mothers of children with ASD and found that exercise is the most effective coping strategy for these mothers. These studies stressed the importance of their own exercise during their leisure time for their health.

Various researchers have identified leisure constraints among parent caregivers of children with disabilities such as increased financial burden, insufficient social support, persistent physical and emotional fatigue, and lack of discretionary time (Mugno et al., 2007; Rivers & Stoneman, 2003). Parent caregivers of children with disabilities feel isolated from other parents who do not have a child with a disability (Corman, 2007). In the context of leisure and recreation, they face challenges in sharing both positive and negative experiences with others because of different circumstances. Moreover, parents who have children with disabilities tend to self-exclude due to perceived feelings of guilt if they engage in leisure activities without their children (Corman, 2007; Norton & Drew, 1994).

## Methods

### Research design

This study adopts a constructivist grounded theory in which “discovering reality arises from the interactive process and its temporal, cultural, and structural contexts” (Charmaz, 2000, p. 524). Grounded theory allows researchers to generate and compare categories and develop relationships among identified themes (Charmaz, 2006). This method enables us to explore leisure behaviours and leisure constraints among mother caregivers who have children with ASD in response to our research inquiry.

### Participants

Purposeful criterion sampling technique was utilized to identify and select study participants. Patton (2002) has suggested this technique allows researchers to capture a certain social phenomenon experienced by study participants and make a valid interpretation of each case. To be eligible for this study, inclusion criteria were: (1) females who were over 18 years of age, (2) those who were able to communicate in English confidently, and (3) those who had children with ASD. The Institutional Review Board (IRB) of our University approved study procedures. Each study participant signed a consent form that outlined the purpose of this study, their rights to withdraw from the study without consequence, and possible benefits and risks of this study.

A total of 12 mothers voluntarily participated in this study. Their age ranged from 37 to 58 years, with a mean age of 45.08 years (median, 44 years). The average age of their children with ASD was 13.92 years. Nine (75%) out of 12 participants were working full or part time at the time the study was completed while three were housewives. Most (83.3%) participants were married at the time the study was completed. Demographic characteristics of study participants are outlined in Table I. This study reached theoretical data saturation based on a guideline suggested by Guest, Bunce, and Johnson (2006).

**Table I:** Characteristics of 12 mother caregivers

Age		
	Average	45.08
	Range	37-58
	Median	44
Working Status		
	Not working	3
	Part time	5
	Full time	4
Marital Status		
	Married	10
	Divorced	2
Number of children		
	Average #	2.33
	Range	1-4
Income		
	Range	20K - 200K
Education		
	High School	3
	Bachelor’s	6
	Master	3

### Data collection

Study participants were recruited via an announcement to mothers of children with ASD to participate in a therapeutic recreation programme, Camp ROCKS, located in the state of Indiana. Those recruited were invited to semi-structured in-depth interviews.These interviews were conducted individually to ensure privacy and allow participants to genuinely express their experiences and perceptions related to involvement in recreation and leisure pursuits.

The research team developed an interview guide based on literature reviews of leisure behaviours and leisure constraints. We applied grand tour and mini-tour interview questions to capture information about leisure behaviours associated with leisure constraints among participants. These questions included the following as examples: “Please tell me what you do in your free time,” “Do you have any challenges when enjoying your free time? If so, please describe them,” and “What resources do you utilize in order to manage stress related to leisure barriers?”

Individual interviews lasted between 40–60 mins. The interviewer made field notes during and after each interview. All interviews were audio-recorded using a Tascam DR-07 Portable Digital Recorder and fully transcribed by two trained students. The audio and written data were transferred to a password-secured computer for data storage and analytical purposes.

### Data analysis

Interview transcripts were analysed with the assistance of Nvivo 11 software to effectively and efficiently manage data. Patterns of commonalities and differences were identified through systematic text condensation method suggested by Malterud (2001). First, after screening scripted materials, units of meaning that represented different aspects of participants’ leisure-related experiences were identified and grouped (open coding). Second, codes for these units were abstracted to identify causal relationships based on the frame of the following elements: phenomenon, causal conditions, context, intervening conditions, action strategies, and consequences (axial coding). Finally, contents of each coded group were summarized to describe patterns and concepts reflecting the most critical aspects of participants’ experiences (selective coding). Main themes that emerged from data analysis were related to participants’ perceptions of attitude, subjective norms, and perceived behavioural control associated with pursing recreation and leisure engagement.

## Findings

Based on patterns in participants’ life experiences and personal statements as they emerged through data analysis, findings were categorized into two sections: (1) leisure pattern, and (2) leisure constraints. Within the leisure pattern section, we identified one theme: leisure negotiation. For leisure constraints, three major themes were identified: (1) lack of time for themselves, (2) constant attention, and (3) difficulty in finding a helper.

### Leisure negotiation

All participants highlighted that they negotiated their leisure choice and preference because of their responsibility and obligation to care for their child with ASD. They were determined to engage in leisure activities based on their child’s leisure interests. They even embraced new leisure activities as a result. They mentioned that supporting their child’s leisure engagement became their new leisure pattern. For example, Julie (42) stated that she mainly focused on whatever recreational activity that her child desired to participate in rather than her own recreational preferences. She said that being involved in summer camp with various recreational programmes for her child became a huge part of her recreational activity. In a similar way, Sarah (42) mentioned that she prioritized her son’s engagement in activities and enjoyed observing her son’s participation. She also said that observing her son’s activities became her main leisure activity.

In the context of leisure and recreation, all participants were aware of unexpected challenges associated with their child’s erratic behaviours such as kicking, screaming, and extreme agitation. They said that they attempted to provide more structured recreational activities and make any necessary adaptation to their engagement such as providing additional adaptive equipment, changing game rules, and modifying materials. Rather than focusing on their own leisure satisfaction, providing effective programmes for their child was their priority. Understanding and studying certain activities became their own primary leisure activity.

Most participants mentioned that they had become more interested in participating in family leisure such as travelling, community-based activities, and outdoor activities with their child as opposed to engaging individually in personally preferred activities. They shared that they were satisfied with their engagement in activities as a family when their child with ASD appeared to enjoy those activities. It appeared that during such leisure engagement, participants often observed their child smiling and expressing happiness. For example, Cathy (58) expressed leisure interest in gardening and fishing because these were her son’s favourite activities. She mentioned that gardening and fishing had not been her preferred activities before. However, she had gained a great interest in them. As a result, she was satisfied with these activities because she was participating in them with her son. Similarly, Julie (42) was involved with church-based activities with her child, although she was not interested in such activities previously. She said that she and her daughter particularly enjoyed being involved in youth group activities and interacting with other youth group members.

Based on these participants’ experiences and perceptions, it was clear that they negotiated their leisure behaviours based on their children’s leisure preferences and choices. They adopted new leisure interests and focused on family-based leisure so that benefits to their children could be maximized. In addition, it appeared that participants sought to provide an effective leisure and recreational setting for their child through efforts such as adapting activities and developing awareness of potential challenges.

### Leisure constraints

All participants experienced various challenges when engaging in leisure and recreational activities. The following three major leisure constraints were identified as themes: (1) a lack of time for themselves, (2) constant attention, and (3) difficult in finding a helper. Each of these constraints posed a significant challenge to participants’ leisure engagement.

#### A lack of time for themselves

One salient theme among identified leisure constraints was mother caretakers’ perception that they had a lack of time for themselves. Participants mentioned that investing their time in personally preferred leisure activities was not realistic because of their role in monitoring and coordinating any participation of their child. Whenever they had free time, they mainly focused on finding leisure resources and therapeutic programmes for their child. Such time investment in obtaining information related to their children’s leisure participation resulted in a lack of time for their own leisure. Young (50), for instance, said that she had been busy searching for and identifying local events, support groups, and recreational opportunities for her child. She spent much of her time coordinating and planning her child’s leisure. As a result, she thought that spending her free time on her own leisure engagement was infeasible.

Most participants mentioned that pursing leisure activities for themselves generated feelings of guilt and shame. Due to such feelings, they invested their time and energy in providing more opportunities for their child instead. One participant, Cathy (58), said:

If I took time for myself and messed up his schedule, I would feel like I’m not giving him the opportunity to grow, and learn, and experience. I don’t want to take away opportunities for him to interact with different people in different situations.

She also mentioned that she was reluctant to engage in leisure activities independently because of the psychological pressure to offer leisure opportunities for her child. Sammy (49) also said that she experienced a lack of time to participate in her preferred leisure activities because of multiple roles and tasks related to caring for her child. She sought to collect as many resources as possible for her child’s engagement in events and therapeutic activities. She mentioned that taking her child to various community-based programmes prevented her from engaging in her own preferred leisure activities.

#### Constant attention

Due to challenges associated with ASD, all participants expressed that they made significant efforts to provide constant care and attention to their child. Such devotion and constant commitment inhibited their participations in personally preferred leisure activities. In addition, when their young adult children with ASD engaged in activities and events, participants said that they were compelled to stay on site and monitor their child’s behaviours. They mentioned that leisure providers often did not know the best strategies to calm their child even though they were generally competent in interacting with individuals with ASD. For example, Jane (41) stated:

Always before we’d go to church camp and I went with her, because I didn’t feel comfortable, I didn’t think it was fair to Lauren or to the other people at the camp for me just to send her when they’re not equipped to deal. You know 95% of the time, she’s going to be fine. 5% she’s going to lose it or something’s going to go wrong, and they don’t understand her or what’s going on to help her. I wasn’t just going to send her off by herself without the proper support in place. So I went with her to camp as the counsellor or cabin leader or whatever.

Her constant engagement in her child’s activities prevented her from pursuing her own leisure activities.

Participants also expressed that, even when socializing with friends in their own leisure time, they were psychologically obligated to check on their child’s safety in a consistent manner. One mother, Ashley (53), said:

I’m scared that he’s going to get out and just start riding his bike. I’m always concerned about strangers, and you know, he doesn’t recognize safety like we do.

She also mentioned that she constantly paid attention to her son’s daily activities both in and out of the home setting. In a similar manner, Sue (44) explained:

I couldn’t go to the beach and take my beach chair and books and let the kids play in the water and just sit and read a book because I constantly have to watch her because she could … especially when she was younger, she had no fear. She’d go out too deep, she would wander over to where some other kids are or whatever with no boundaries, it’s like she’d walk to the other side of the lake, you know?

Due to her concern for her child’s safety, Sue provided constant attention and care. As a result, she could not concentrate on her own leisure pursuits.

#### Difficulties in finding a helper

All participants expressed that they had difficulty in finding assistants such as sitters that would give them free time to enjoy their personally preferred leisure activities. Participants explained that while family members such as spouses, parents, and other relatives were typically a reliable source for taking care of their child, they were not often available when needed. They also shared similar experiences related to the difficulty in finding reliable and knowledgeable sitters for their child. Cathy (58), for example, expressed that a former sitter had a difficult time in handling unexpected situations when her child exhibited erratic behaviours and deficient communication skills. She stated:

I tried [that type of] care several years ago, and that was supposed to be someone coming into the home, being with Alex while I did on my own thing. It was terrible … most people that I’ve been involved with through healthcare services or community rehab services don’t have the knowledge base to interact with an autistic person … they don’t know how to engage in that comfort level.

She also pointed out that her former sitters had difficulty communicating with her son. Such failure of these services meant she was unable to engage in preferred leisure activity independently.

Another mother caretaker, Susan (39) said that she received negative comments and feedback from a recreation programme director because of her child’s deficient social skills and communication skills. She said:

I did try to put him in the After-School Programme at the YMCA this Spring semester … that did not go very well with him. He had a good time, but the staff, they weren’t trained to deal with kids like Caeden … I ended up taking him out, because I just got tired of hearing the complaints that it was an inappropriate behaviour … so my dad started watching him after school the two days that I went [to class].

Susan expressed frustration when she acknowledged there was a lack of competent providers for her child. Similarly,Julie (42) shared a similar experience that her child expressed anger and aggression when she was not around her. Julie said that only her mother was a reliable and knowledge caretaker of her child. Besides her mother, Julie expressed, it was difficult to find a reliable and knowledge person who could comfort her child and interact with her effectively.

## Discussion

This qualitative study examined leisure behaviours associated with leisure constraints experienced by parent caregivers who have a child with ASD. This study found that participants modified their leisure patterns as they sought to provide more recreational opportunities for their children. Our findings indicated that participants negotiated their leisure choices and developed family adaptability related to family leisure to suit desires and needs of their child with ASD. In particular, providing necessary care and support for their child served as a major inhibitor of their own personal leisure engagement. Finding a way to more effectively support parents to provide recreational opportunities for their children and more effectively care for those children will be critical if leisure service providers and recreational therapists wish to increase leisure engagement and satisfaction for these parent caretakers.

Prior studies have demonstrated that parent caregivers who have children with disabilities focus mainly on their children’s life opportunities and engagement rather than on themselves (Abbeduto et al., 2004; Weiss, 2002). Findings of this study support the idea that mothers of children with ASD would reorganize their priorities to provide their children with a safe and healthy leisure environment and equip them with skills and resources related to leisure. Such emphasis on their children’s opportunities above their own allowed these participants to develop resiliency and adaptability in negotiating their leisure behaviours and leisure choices. However, it also significantly prevented them from engaging in their own preferred leisure activities independently.

Family leisure has been found to increase positive family functioning, to increase quality of life for the family. It contributes to stress management and coping skills (Mactavish et al., 2007; Townsend & Van Puymbroeck, 2017). In our study, participants expressed that they engaged in various forms of family leisure and often embraced them as their new leisure activities. These findings indicated that participants had positive interactions with their children and spent quality time together through these shared leisure pursuits. This suggests that involvement in family leisure can play an essential role in family wellbeing and family functioning. On the other hand, findings of this study suggest that obligation to family leisure can serve as an additional barrier that prohibits parent caregivers from participating in their own preferred leisure activities.

Roach, Orsmond, and Barratt (1999) have suggested that parents who have children with developmental disabilities will develop resilience and adapt to the special demands of their children. They also indicated that parents with children having developmental disabilities experienced high levels of stress, constraints, and challenges. This study suggested that participants developed an ability to negotiate their own leisure engagement and leisure constraints. As a result, participants could manage challenges and constraints related to special demands of their children. This finding suggests that any individual can reshape and form new patterns of leisure engagement to fit their life circumstances.

Various scholars have stressed the importance of inclusive leisure and recreation for individuals with disabilities (Modell & Imwold, 1998; Piatt & Jorgensen, 2011). Their studies have suggested that service providers should focus on offering inclusive leisure programmes designed to promote cross-cultural contact among individuals with and without disabilities. Our findings indicate that, despite such suggestion, leisure service providers may lack multicultural competence and training in working with individuals with disabilities. This lack of skills and competency among leisure service providers seems to have served as a significant leisure constraint to participants. Thus, this should be rectified.

Multiple studies have indicated that lower income and education levels play an important role in leisure constraints as a structural element (Alexandris & Carroll, 1997; Shores, Scott, & Floyd, 2007). Such leisure constraints can negatively affect life satisfaction, leisure satisfaction, health, and wellbeing (Chick, Hsu, Yeh, & Hsieh, 2015; Spiers & Walker, 2009). This study reinforces the idea that caregivers encountering similar leisure constraints may experience low life satisfaction and quality of life. This suggests that minimizing factors that impede leisure engagement among parent caregivers might be important for their health.

### Implications and conclusion

Findings of this study indicated that participants—mothers of children with ASD—encountered various leisure constraints that might negatively affect their perception of health and wellbeing. To counter these negative effects, leisure service providers and recreational therapists need to design and implement leisure education programmes for mother caregivers. Leisure education has been defined as “an individualized and contextualized process through which a person develops an understanding of self and leisure education and identifies and learns the cluster of skills necessary to participate in freely chosen activities that lead to an optimally satisfying life” (Bullock & Mahon, 1997, p. 381). Leisure education programmes can help participants expand and utilize their leisure resources, develop leisure and recreation skills, and improve the value and appreciation of leisure (Kleiber, 2001; Mundy, 1998). Therefore, a variety of leisure education programmes that can offer rich opportunities for parent caregivers are needed to increase their leisure awareness and leisure resources.

Findings of this study also suggest that leisure service agencies and organizations need to provide training programmes for their staff members. Through training programmes, leisure service providers can gain knowledge and information related to certain disabilities and build confidence in designing programmes and interacting with children with disabilities such as ASD so that parents could pursue their own leisure interests. In addition, as part of such training programmes, providers should communicate with parents who have a child with a disability so that they understand what to expect regarding potential challenges.

Lastly, certain recreation events such as summer and winter camps pose a unique opportunity to meet the needs of parents of children with disabilities alongside their children. Researchers and practitioners often collaborate with each other to develop leisure and recreational programmes for children with ASD. They should do the same for parents of these children. Findings of this study demonstrate a significant need to develop special programmes to serve both parent caregivers and their children with a disability independently but congruently so that parents may obtain the same benefits of leisure participation that their children often get.
